# Role of Obesity, Physical Exercise, Adipose Tissue-Skeletal Muscle Crosstalk and Molecular Advances in Barrett’s Esophagus and Esophageal Adenocarcinoma

**DOI:** 10.3390/ijms23073942

**Published:** 2022-04-01

**Authors:** Jan Bilski, Monika Pinkas, Dagmara Wojcik-Grzybek, Marcin Magierowski, Edyta Korbut, Agnieszka Mazur-Bialy, Gracjana Krzysiek-Maczka, Slawomir Kwiecien, Katarzyna Magierowska, Tomasz Brzozowski

**Affiliations:** 1 Department of Biomechanics and Kinesiology, Chair of Biomedical Sciences, Faculty of Health Sciences, Jagiellonian University Medical College, 31-008 Cracow, Poland; agnieszka.mazur@uj.edu.pl; 2 Department of Physiology, Faculty of Medicine, Jagiellonian University Medical College, 16 Grzegorzecka Street, 31-531 Cracow, Poland; monika.pinkas@doctoral.uj.edu.pl (M.P.); dagmara1.wojcik@uj.edu.pl (D.W.-G.); m.magierowski@uj.edu.pl (M.M.); edyta.korbut@uj.edu.pl (E.K.); gracjana.krzysiek-maczka@uj.edu.pl (G.K.-M.); slawomir.kwiecien@uj.edu.pl (S.K.); katarzyna.magierowska@uj.edu.pl (K.M.)

**Keywords:** esophageal adenocarcinoma, Barrett’s esophagus, gastroesophageal reflux disease, obesity, adipose tissue, adipokines, inflammation, myokines, exercise

## Abstract

Both obesity and esophageal adenocarcinoma (EAC) rates have increased sharply in the United States and Western Europe in recent years. EAC is a classic example of obesity-related cancer where the risk of EAC increases with increasing body mass index. Pathologically altered visceral fat in obesity appears to play a key role in this process. Visceral obesity may promote EAC by directly affecting gastroesophageal reflux disease and Barrett’s esophagus (BE), as well as a less reflux-dependent effect, including the release of pro-inflammatory adipokines and insulin resistance. Deregulation of adipokine production, such as the shift to an increased amount of leptin relative to “protective” adiponectin, has been implicated in the pathogenesis of BE and EAC. This review discusses not only the epidemiology and pathophysiology of obesity in BE and EAC, but also molecular alterations at the level of mRNA and proteins associated with these esophageal pathologies and the potential role of adipokines and myokines in these disorders. Particular attention is given to discussing the possible crosstalk of adipokines and myokines during exercise. It is concluded that lifestyle interventions to increase regular physical activity could be helpful as a promising strategy for preventing the development of BE and EAC.

## 1. Introduction

Esophageal cancer is currently a major public health problem in Europe and worldwide due to its aggressive nature and low survival rate. There are two main histological types of esophageal cancer, squamous cell carcinoma (SCC) and adenocarcinoma (EAC), which differ significantly in their patterns of etiological factors. Risk factors for SCC include male gender, alcohol consumption, smoking, certain dietary factors, and poor oral hygiene [1]. Obesity, especially gastroesophageal reflux disease (GERD), male gender, and smoking are major risk factors for EAC [1,2,3,4,5]. Interestingly, EAC is the most deadly and fastest growing cancer in the United States. The presence of GERD is associated with an increased risk of developing Barrett’s esophagus (BE), a precancerous condition characterized by the replacement of normal squamous epithelium with a columnar cylindrical epithelium, usually with intestinal metaplasia [6]. As these risk factors are at least partially modifiable, there are many options for intervention to prevent EAC. As BE is the only known precursor to EAC, advances in the monitoring of BE are essential to enable diagnosis and improve patient outcomes.

## 2. Epidemiology

The incidence of EAC has rapidly increased in Europe and the United States over the past four decades and even more than six-fold since the 1970s, surpassing SCC [5,7,8,9,10]. Although before the 1970s, EAC was less common than SCC, it now has the fastest growing incidence of any type of cancer in Western populations [11,12,13,14,15,16,17]. EAC is also becoming an increasingly common cause of morbidity and mortality in the United States and Europe [5]. The incidence of EAC is still predicted to increase significantly in the coming years [8].

The reason for this increase is unclear, being at least in part attributed to the concomitant increase in the incidence of GERD and obesity worldwide [16,18]. As shown in a recent systematic review, the incidence of GERD was 18.1–27.8% in North America and 8.8–25.9% in Europe [19]. The physiological reflux of gastric contents into the esophagus occurs in most individuals, but when these episodes occur regularly, they lead to GERD [20]. Patients with GERD show an increased risk of developing BE [21], a precancerous condition defined by the replacement of normal squamous epithelium with a columnar epithelium, usually accompanied by the intestinal metaplasia. These conditions can lead to low-grade dysplasia, high-grade dysplasia, and ultimately to EAC development [5,9,22,23,24,25,26]. BE is associated with a 10- to 40-fold increased risk of EAC development [27,28,29]. BE patients had a higher body mass index (BMI) than the control group and more often presented a record of smoking and reported recurrent GERD symptoms [30]. Changes in lifestyle and eating habits, such as a sedentary lifestyle or lack of physical activity, as well as dietary aspects mainly involving a high-fat diet (HFD), are epidemiologically correlated with the development of EAC [31,32]. Obesity, with its epidemic proportions around the world, is a major clinical and public health problem of widespread importance [33,34]. As mentioned earlier, obesity may contribute to GERD pathogenesis, but it is also considered an important risk factor for cardiovascular disease, stroke, type 2 diabetes, high blood pressure, osteoarthritis, liver disease, chronic kidney disease, and several types of cancer [34,35,36,37,38].

Epidemiological studies have shown that obesity and the incidence of EAC are similar, and there is a strong correlation between obesity and the risk of EAC [39,40,41,42,43,44,45,46,47]. Obesity constitutes a significant risk factor for developing BE as well as EAC [2,48], and this increased risk of these disorders is primarily linked with visceral obesity [49].

As stated by the World Health Organization [50], obesity is defined as an excessive or abnormal accumulation of body fat that has a negative impact on health. In order to determine the total amount of fat in an organism and the distribution of fat in an organism, a variety of methods have been used, including anthropometric measures such as body mass index (BMI), waist circumference, waist-to-hip ratio (WHR), bioelectrical impedance analysis, dual-energy X ray absorptiometry (DEXA), computed tomography (CT) scan, and magnetic resonance imaging (MRI) [51].

The most commonly used measure to diagnose and classify obesity is the BMI, defined as a person’s weight in kilograms divided by the square of their height in meters (kg/m^2^). BMI values from 25 kg/m^2^ to 29.9 kg/m^2^ is considered overweight and higher values of BMI are defined as obesity [47,52].

Significant associations between BMI and EAC risk have been found with positive BMI value–response relationships [39,46], with the highest risk occurring among those with the most severe obesity [39]. The retrospective cross-sectional study has shown that being overweight is estimated to entail a 2.5-fold increased risk of BE [52]. Another study demonstrated a relationship between BMI and the length of BE mucosa [53]. Many studies indicate that exclusive reliance on the BMI as an indicator of obesity is a significant limitation in research designed to determine the relationship between obesity and human diseases [54,55,56,57,58]. The BMI consists of adipose tissue, which represents the mass of skeletal muscles, bones and organs, and the lean mass index, which is the sum of peripheral and visceral fat (VAT) [59]. All these components of BMI play different roles in influencing the health of the human body. While these epidemiological studies are very important, they will not be completely accurate. Most epidemiological studies use obesity measures, such as BMI, that do not reflect the real picture and pathomechanism of obesity. It should also be considered that it is very difficult to separate a relative contribution of diet and obesity to cancer development [60]. These factors may act independently or in combination, influencing, for example, the composition of the microbiota in the intestine [61]. The importance of this problem is demonstrated by individuals with a normal-body-weight metabolic obesity, who present with metabolic disorders and an increased risk of several obesity-related cancers, even though they look thin [62]. Waist circumference (visceral obesity indicated by a waist circumference >94 cm in men and >80 cm in women) and WHR (>0.9 for men and >0.8 for women) are two methods of determining visceral obesity.

The imaging techniques and EXA provide more accurate adipose tissue volume and distribution estimations. When the higher mortality risk is linked to an increase in total adipose tissue assessed by DEXA, the imaging techniques have indicated that fat distribution (specifically visceral adipose tissue) rather than overall fat levels are more predictive. For a long time, the term “visceral obesity” was used to describe an excess of fat in the abdominal cavity. However, it is now widely accepted that VAT comprises fat deposits found throughout the body, including the omentum, mesenteric, epiploic, gonadal, epicardia, and retroperitoneal depots, and is frequently accompanied by other ectopic fat deposits. When reviewing the literature, it becomes clear that studies that present normative VAT measurements are scarce [63]. Recently, Elliot et al. [64] examined the visceral fat area (VFA) in patients with EAC using computer tomography and defined visceral obesity as VFA > 163.8 cm^2^ for men and 80.1 cm^2^ for women, respectively.

Regarding esophageal disorders, it is important to note that the esophago-gastric junction (GC) fat pad, which envelopes the distal esophagus and shares its vascularity, warrants specific attention in this context [65]. In obesity, the EGJ fat pad is the location of VAT accumulation and source of proinflammatory and pro-cancerous substances, as Paris and colleagues pointed out in a recent paper [66].

Visceral obesity is a risk factor for BE and EAC, regardless of BMI, and its effects are influenced by reflux-dependent and reflux-independent mechanisms [3,49,67]. For instance, patients receiving cholesterol-lowering statin therapy have shown a reduced incidence of BE [68,69] and EAC [70,71,72]. Numerous studies have confirmed that central abdominal obesity and increased visceral fat rather than BMI have been postulated to constitute a significant risk factor for EAC and BE [73,74,75,76,77]. It has been suggested that the influence of obesity on the risk of BE and EAC may be underestimated in studies based solely on BMI [32,78]. Kramer et al. observed no correlations between BMI, WHR, and short-segment BE, but they did find a statistically significant correlation between WHR and long-segment BE in their study [79]. However, waist circumference and WHR are non-specific measures of abdominal fat, which has a visceral and a subcutaneous component. It has been shown that the relative distribution of abdominal fat in these two compartments has a varied effect on the risk of BE. Abdominal cavity CT showed that BE is associated with abdominal visceral obesity [73]. The study revealed that VAT, but not SAT, is related to BE [12]. White men with BE have a high VAT/SAT (VAT/SAT) ratio, which has been linked to BE, and this association between VAT to SAT and BE remains even when there is no evidence of GERD [73].

Barrett’s esophagus has been arbitrarily divided into a long (≥3 cm long) and a short (<3 cm long) segment [80] and this division is of clinical relevance also in the context of obesity. Some research investigated whether the link between visceral obesity and BE differed for the long segment vs. the short segment [73,79]. They discovered that the association between abdominal obesity as evaluated by WHR [79] or the VAT/SAT ratio as determined by CT [73] is especially prevalent in patients with long-segment BE who are white men. An association has also been demonstrated between the metabolic syndrome and BE and EAC, and interestingly, this relationship is stronger for men and, for them, these effects appear to be independent of previous GERD history [81,82].

The dietary factors that have been identified as possible risk factors for BE and EAC do not have to be restricted to HFD. Diet may impact the risk of developing EAC via an impact on the risk of developing GERD or BE, as well as the rate at which the patient progresses from BE to EAC. Kubo et al. observed that eating a diet rich in fruits and vegetables and fish was inversely related to the risk of BE, but the Western diet was associated with an increased risk of BE. The consumption of red meat was found to be positively linked with EAC development [83]. Diet may influence the risk of developing EAC via an impact on developing GERD or BE and the rate at which the patient progresses from BE to EAC. Kubo et al. [84] observed that eating a diet rich in fruits and vegetables and fish was inversely related to the risk of BE, but the Western diet was associated with an increased risk of BE. In their research, Jiao and colleagues [85] confirmed that higher consumption of red meat and saturated fat may be associated with an increased incidence of BE. They postulated that this effect could be explained by advanced glycation end products (AGEs), found in high concentrations in high-fat foods and high-temperature cooked meat.

Larger consumption of dark green vegetables, on the other hand, was related to a decreased risk of BE, as proven by the same group of investigators [86]. They hypothesized that this protective effect might be explained by many nutritional components, including fiber, antioxidants, and folate [86].

## 3. The Role of Obesity in BE and EAC Development

Obesity is considered a metabolic disease causing a chronic, low-grade inflammation called meta-inflammation, characterized by the activation of pro-inflammatory pathways, and resulting in an increase in the synthesis of acute-phase reagents, such as C-reactive protein, and the production and release of pro-inflammatory cytokines [47,87]. Adipose tissue is not homogeneous; there are two main types: white adipose tissue (WAT) and brown adipose tissue (BAT) [88]. WAT is made up of adipocytes embedded in a collagen skeleton; in addition to adipocytes, adipose tissue contains a subpopulation of stem cells called stromal vascular fraction cells, preadipocytes, fibroblasts, leukocytes, macrophages, and endothelial cells.

Anatomically, the WAT consists of two major compartments: subcutaneous adipose tissue (SAT) and visceral adipose tissue (VAT), each with its own metabolic and immunological characteristics [89,90]. VAT and SAT can store energy in the form of triacylglycerols. Both can produce physiologically active substances that affect energy balance and metabolism. Additionally, the visceral WAT layer protects the body’s essential organs, while the subcutaneous WAT layer acts as an insulator against heat and cold [88].

The metabolic consequences of obesity are strongly influenced by differences in fat distribution across the body. Premature mortality and a higher incidence of metabolic and cardiovascular diseases are all linked to visceral obesity. However, those who store WAT mostly subcutaneously, on the other hand, have a lower risk of death and metabolic diseases. In particular, VAT shows increased pro-inflammatory and pro-cancer properties compared to SAT, and its hypertrophy has been associated with a pro-inflammatory state [87,91].

Healthy VAT is well-vascularized, with regulatory and immunosuppressive cells and the production of anti-inflammatory molecules [92]. As a result of the VAT accumulation in obese persons, the pro-inflammatory transformation occurs, accompanied by the production of several pro-inflammatory substances by adipocytes. With the development of visceral obesity, adipocytes become hypertrophic and hypoxic, and eventually die, triggering an innate immune response [92]. Reduced production of anti-inflammatory adipokines such as adiponectin (APN) is also characteristic of hypertrophic adipocytes. The inflammatory cells’ infiltration of adipose tissue further increases the production of inflammatory mediators [92,93,94].

With extensions to the sub-scapular, cervical, and axillary areas, one may find the bulk of the BAT depot in the deep interscapulum region. However, BAT can also be found at aortic, paraspinal, and adrenal sites. Adaptive thermogenesis is the primary role of BAT’s multicellular, mitochondria-rich, and uncoupling protein 1-positive adipocytes. Obesity and insulin resistance are negatively related to the amount of active BAT in the body [94].

A new type of brown adipocytes embedded in the WAT, referred to as beige or brite cells, has been discovered in recent years. These cells are activated in response to cold, β-adrenergic stimulation, and peroxisome proliferator-activated receptors (PPAR-), a process known as adipose tissue browning [95]. Classic brown adipocytes derive from MYF5+ (muscle developmental gene) mesenchymal stem cells in the embryonic mesoderm, whereas beige cells appear to arise from endothelial and perivascular cells in WAT stores [96]. In humans, the MYF5 gene encodes a protein known as myogenic factor 5 that regulates muscle differentiation or myogenesis, both of which are essential for the development of skeletal muscle.

WAT and BAT can communicate with other organs to control metabolism by secreting adipokines and batokines, respectively, signaling lipid types (lipokines), and exosomal microRNAs (miRNAs) [84,85]. Adipokines and batokines, signaling types of lipids (lipokines), and exosomal microRNAs (miRNAs), all of which are released by WAT and/or BAT, act as mediators for inter-organ communication and can regulate metabolism [97,98]. WAT in particular acts as a hormonal organ that produces biologically active adipokines, such as APN, interleukin (IL)-1, IL-6, IL-8, interferon-γ, TNF-α (tumor necrosis factor-α), leptin apelin, chemerin, and resistin. Adipokines can regulate metabolic homeostasis and influence immune function [99].

Obesity is a well-defined risk factor for several cancer types and is associated with poorer outcomes [100]. Several hypotheses explain how obesity might contribute to EAC development and growth.

**Figure 1:**
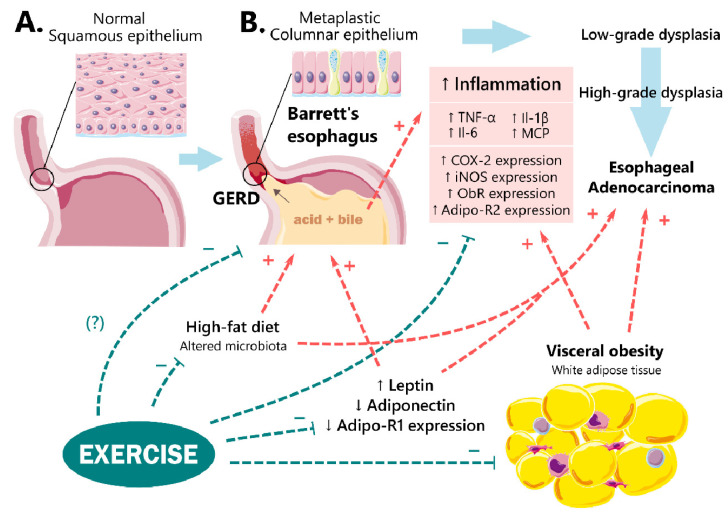
Normal esophageal squamous epithelium reflecting healthy esophagus (**A**) and hypothetical model of gastroesophageal reflux disease (GERD) pathogenesis leading to Barrett’s esophagus (BE) and subsequent progression to esophageal adenocarcinoma (EAC) (**B**) in obese patients; isolated BE cell lines in vitro or experimental animal models of diet-induced obesity. The inflammatory molecular changes associated with the development of BE include changes in the molecular expression of pro-inflammatory factors such as the upregulation of COX-2, iNOS, ObR, and Adipo-R2, followed by the downregulation of adiponectin and Adipo-R1 at the mRNA level and/or and an increase in the level of pro-inflammatory cytokines (TNF-α, IL-1β, and IL-6) and chemokines (MCP) in plasma and esophageal tissues. Exercise can reduce the inflammatory effect of GERD and possibly the number of GERD episodes by exerting an anti-inflammatory effect by reducing the esophageal expression and plasma levels of proinflammatory factors and cytokines, restoring the leptin-to adiponectin-ratio, altering the gut microbiota, and counteracting visceral obesity exacerbating GERD, BE, and then EAC.

The pathomechanism by which VAT promotes EAC is not clear, but it is now generally accepted that abdominal obesity mediates its influence via both mechanical and metabolic effects. The most apparent mechanism seems to be the worsening of GERD due to mechanical factors [101]. Although obesity, primarily abdominal, is a significant contributor to the development and severity of GERD and BE, it is also an independent risk factor for EAC, with a 52% increase in risk for every five BMI units [40,101]. These observations indicate the existence of “GERD-independent”, possibly metabolic mechanisms mediated by VAT in the development of esophageal cancer in obese individuals (Figure 1) [6,102].

### 3.1. Obesity and GERD

A key relevant pathway linking obesity with EAC could be the occurrence of GERD, as long as the severe GERD is associated with an up to 40-fold increased risk of EAC [103]. GERD is a global disorder and unquestionably a disease that is directly linked to obesity [24]. Obesity triples the chance of developing GERD [104] and doubles the risk of erosive esophagitis [105]. Moreover, the prevalence of GERD is proportional to the severity of obesity [106]. The prevalence of GERD symptoms in patients with morbid obesity reaches 50% [107]. Successful weight loss and the therapy to reduce the visceral adipose tissue have been shown to significantly reduce GERD symptoms [108,109,110,111,112,113].

Several mechanisms may be responsible for such a strong link between obesity and GERD. The already recognized mechanism of exacerbation of GERD is attributed to mechanical factors, i.e., disruption of the reflux barrier of the gastroesophageal junction [101]. Abdominal obesity is strongly associated with a higher number of transient lower-esophageal sphincter relaxations and abnormal peristalsis [114,115,116,117,118]. Impaired esophageal motility could also lead to abnormal esophageal clearance [114,115]. Abdominal obesity may predispose to disruption of the anatomic gastroesophageal junctions, causing hiatal hernia formation [106,119].

Abdominal obesity may lead to an increased intra-abdominal pressure [120]. Recently, Del Grande et al. [121] reported that the presence and severity of GERD in obese subjects were positively correlated with the trans-diaphragmatic pressure gradient caused by increased intra-abdominal pressure due to visceral obesity. It is of interest that these effects were recorded independently of BMI. Dietary habits such as irregular food consumption, especially in the evening hours, and a diet rich in reflux-promoting factors may also worsen GERD symptoms in obese subjects [122,123,124,125,126,127,128].

It is not excluded that adipokines released by adipose tissue may influence the development and severity of GERD [107]. Increased leptin and leptin receptor (ObR) levels and decreased APN levels were reported in obese patients with GERD [129,130,131,132], and increased leptin levels, widely considered as a marker of obesity, have been associated with frequent GERD symptoms [133] and clinical and endoscopic severity of GERD [130]. Numerous studies have revealed that the serum APN levels are inversely associated with BE in GERD patients [134,135,136].

In obese subjects, pro-inflammatory cytokines and adipokines released from pathologically altered VAT may play a role in the development of esophagitis [137]. For example, Murata et al. [138] showed that administration of leptin worsened reflux esophagitis in rats with evident infiltration of CD3 + T cells and a significant increase in the levels of macrophage inhibitory factor (MIF) and TNF-α, IL-1β, and IL-6 in esophageal tissue, the latter being considered as the downstream targets of MIF. Interestingly, abdominal visceral fat and leptin, independently of each other, increased the risk of reflux esophagitis [137]. Moreover, the decreased APN levels were correlated with an increased risk of erosive esophagitis observed in a large group of more than two thousand patients undergoing physical check-up [139].

### 3.2. Role of Adipokines

Chronic inflammation associated with epithelial metaplasia, present in BE, increases cancer risk, presumably favoring the tumor microenvironment and tumor progression [140,141,142]. In a pathologically modified VAT, the release of increased amounts of the pro-inflammatory cytokines [143,144] and an enhancement of plasma levels of pro-inflammatory adipokines in BE patients have also been observed [135,145,146,147,148,149,150].

In obese conditions, pathologically modified adipose tissue demonstrates an altered signalling molecules profile, forming a pro-tumorigenic milieu [60,151]. The low-grade chronic inflammation develops due to the adipose tissue in obesity. The pro-inflammatory transformation associated with the pathological expansion of visceral fat and infiltration of adipose tissue by inflammatory cells prompts the secretion of many pro-inflammatory mediators from adipose tissue [93]. Adipocytes secrete adipokines such as leptin and TNF-α that may exhibit mitogenic effects resulting in the progression of BE to EAC. Adipokines and other pro-inflammatory cytokines may promote carcinogenesis through various mechanisms [152]. Adipokines can act locally and exert a systemic endocrine effect or exhibit a tumorigenic effect [60]. Inflammatory cells infiltrating obese adipose tissue can also produce reactive oxygen species (ROS) known for their mitogenic effects at low concentrations, thus playing the role of tumor promoters [153]. This systemic and chronically elevated secretion of pro-inflammatory cytokines and ROS in obesity may undoubtedly promote carcinogenesis [154].

Currently, the impact of obesity on the tumor microenvironment (TME) is of great interest [60]. The TME is composed of cellular components such as endothelial cells, immune cells such as microglia, granulocytes, lymphocytes, macrophages, and tumor stromal cells, including stromal fibroblasts and non-cellular components of the extracellular matrix [60]. Low-grade fat inflammation in obesity is comparable to that of the TME, clearly suggesting the ability of altered adipose tissue to stimulate tumor growth [103,155]. Data from animal models indicate that pro-inflammatory cytokines are essential for the development of BE [156]. Leptin has been shown to increase the proliferative and invasive capacity of Barrett cell lines, and APN has been shown to block the cancer-promoting effects of leptin in experimental models [157,158,159,160,161,162,163,164]. When EAC cell lines were cultured in adipose tissue conditioned medium with VAT, their increased ability to proliferate, migrate, and invade was observed [165].

#### 3.2.1. Leptin

Leptin [166] was the first adipokine to be described, and its plasma levels increase in proportion to adipose tissue mass. Leptin is a pro-inflammatory adipokine that has been shown to contribute to the local and systemic inflammatory milieu in obesity via a mechanism involving the activation of pro-inflammatory cells, the stimulation of the Th1 cell response, and the production of pro-inflammatory cytokines [167]. In addition, this peptide can exert a direct effect on esophageal epithelial cells, influencing subsequent stages of the EAC cascade [164,168,169]. Recent studies have shown that leptin inhibited apoptosis and increased proliferation in obesity-related cancer cell lines [170,171,172], including EAC [173]. For instance, Ogunwobi et al. [173] showed that leptin stimulated proliferation and inhibited apoptosis via extracellular signal-regulated kinase, p38 mitogen-activated protein kinase, phosphatidylinositol 3′-kinase/Akt, and Janus tyrosine kinase 2-dependent activation of cyclooxygenase-2 and prostaglandin E2 production in OE33 cells (Barrett’s-derived EAC line). These investigators have suggested that these pathways may link obesity with the development of EAC [173]. Human studies have revealed that leptin ObR is abundantly expressed in precancerous (BE) and cancerous (EAC) conditions [174,175]. Elevated serum leptin levels have been considered as an independent risk factor for BE development [145,158,159,163,164,176,177]. In BE patients, elevated levels of leptin and insulin resistance were associated, independently of GERD, with an increased risk of EAC, while elevated levels of high-molecular-weight APN were inversely correlated with EAC [163].

#### 3.2.2. Adiponectin

APN is an adipokine whose plasma levels, unlike leptin, decline as body fat increases. This adipokine was reported to improve insulin sensitivity and to exert anti-inflammatory and anti-tumor effects [178]. Low APN levels are an independent risk factor for several cancers [179,180,181], including EAC [160,161,162]. Earlier studies documented that patients with BE and EAC had significantly lower levels of the anti-inflammatory adipokine, APN, compared to healthy controls [160,161,162]. Moreover, APN reduced the leptin-induced proliferation in EAC cells by acting through the APN type 1 receptor [182], inhibiting leptin-induced signalling and the procarcinogenic potential of this peptide by activating protein tyrosine phosphatase 1B, and thus, alleviating early events in leptin-induced signal transduction [157].

### 3.3. The Role of Insulin Resistance

Another mechanism by which the overgrowth of VAT may influence the development of EAC is the induction of insulin resistance. Epidemiological studies have shown that patients with metabolic syndrome have a higher incidence of cancer [152]. Hyperinsulinemia is a significant risk factor for the development of BE [177] and patients with BE and insulin resistance have an increased risk of developing EAC [163]. The reduced tissue sensitivity to insulin resulted in an increase in glucose and insulin levels, and chronic hyperinsulinemia promoted the secretion of insulin-like growth factor 1 (IGF-1) and a decrease in the production of IGF-binding proteins [183]. Insulin itself may be mitogenic and anti-apoptotic, but IGF-1 is likely to mediate most of the proliferative effects of insulin [183]. The increased expression of IGF-1 receptors is strongly associated with malignant progression of BE to EAC [184]. Interestingly, IGF-1 significantly stimulated the proliferation in EAC cell lines and the serum IGF-1 levels were elevated in patients, with EAC being further potentiated in patients with visceral obesity as compared with non-obese individuals [185]. Moreover, the IGF-1 receptor expression in dissected EAC tumor samples was significantly higher in patients with visceral obesity than the non-obese patients. Survival was longer in patients without expression of the IGF-1 receptor than in patients with IGF-1-receptor-positive tumors [186].

In a mouse model, hyperinsulinemia significantly increased the incidence of esophageal cancer in the presence of duodenal reflux, and both the insulin receptor and IGF1 receptors were overexpressed [187]. The hypotheses for adipokines and insulin resistance in obesity in the context of BE and EAC pathogenesis may overlap because insulin resistance is at least partially mediated by adipokines and cytokines released from the altered adipose tissue [154].

### 3.4. Role of Diet

The potential effect of HFD feeding on the development of BE and EAC has been investigated in experimental animal models. In a rat model of BE, Clark et al. [188] observed that reflux of gastroduodenal content into the lower esophagus of rats could induce both Barrett’s metaplasia and EAC, and an HFD promoted carcinogenesis. Chen et al. [189] demonstrated that HFD intake changed the bile-acid composition of bile juice and enhanced the development of BE and EAC via an increase in the concentration of taurine conjugates in bile juice in a rat duodenal-contents reflux model. Molendijk et al. observed that HFD increased the severity of inflammation and the length of esophageal metaplasia [190]. Feeding a HFD in rodents was correlated with more proliferative EAC tumors associated with alterations in the secreted adipokines profile [191]. HFD feeding accelerated carcinogenesis in a mouse model of BE by altering the gut microbiota, independently of obesity [192].

The effect of HFD on the esophageal microbiota and its role in the development of EAC has been recently investigated [193,194]. The distal esophagus has a characteristic microbiome mainly composed of the oral flora that changed in the BE and GERD [195,196,197]. Furthermore, there is already sufficient evidence to believe that the esophageal microbiota is involved in the EAC cascade at different stages of tumorigenesis [193,194,195,196,198,199,200,201].

## 4. Role of Physical Activity

The observations presented above suggest that therapies aimed at improving the endocrine profile of adipose tissue may translate into practical clinical interventions. Past studies have shown the benefit of alternative non-pharmacological interventions such as exercise in the treatment of several chronic diseases including cardiovascular and metabolic diseases as well as cancer [202,203,204]. Epidemiological studies showed that regular physical activity may prophylactically reduce the risk of developing cancer, as well as influencing cancer activity and its progression. Physical activity has been shown to have a beneficial effect on cancer therapy because physical activity significantly reduced the risk of developing various types of cancer [205,206,207].

Although acute, vigorous exercise can induce gastroesophageal reflux disease, moderate and regular exercise is associated with a reduced incidence of erosive oesophagitis [208]. Data from a prospective study in the Norwegian population showed a significant protective effect of regular physical activity [209]. Another study found that people with reflux symptoms were less physically active than those without symptoms [210]. Interestingly, monozygotic twins who were less physically active showed the typical symptoms of GERD compared to those who exercised regularly [211]. Regular physical activity helps to maintain a healthy body weight, thus reducing the risk of obesity-related GERD [212]. Regular exercise is also beneficial in preventing reflux by strengthening the crural diaphragm, an essential component of anti-reflux mechanisms [209].

A recent epidemiological study from Germany showed that BE patients were more likely to be physically inactive and had a higher percentage of poor performance indicators than controls [213]. The relationship between physical inactivity in humans and the risk of developing EAC is relatively well established, but the mechanism by which exercise can improve human outcomes for BE and EAC is poorly understood. For example, an association between a sedentary lifestyle and an increased risk of EAC has been documented [205,214]. Moreover, recent meta-analyses, reviews, and epidemiological studies highlight the importance of physical activity in reducing the risk of EAC by a mechanism that may be associated with a reduction in the release of pro-inflammatory and carcinogenic adipokines [205,214,215,216,217,218,219,220].

### Role of Adipose Tissue-Muscle Crosstalk

The exact mechanisms by which exercise protects against chronic diseases such as BE and EAC remain unknown, but they can be attributed not only to weight management through exercise, but also to exercise-inducing anti-inflammatory and antioxidant effects. Myokines, which are substances generated and released by skeletal muscle, may be responsible for the anti-inflammatory benefits of moderate exercise, whereas high-intensity exercise can lead to inflammation and immunosuppression [221,222,223]. A growing number of myokines have been identified, including interleukin-6, interleukin-8, and interleukin-15, brain-derived neurotrophic factor, ciliary neurotrophic factor, vascular endothelial growth factor, fibroblast growth factor 21, irisin, meteorin-like, and aminoisobutyric acid (BAIBA), secreted protein acidic and rich in cysteine (SPARC) [196], and oncostatin-M (OSM) [224].

Hence, physical exercise may exert its anti-inflammatory effect via a decrease in VAT and the generation of an anti-inflammatory environment with each bout of exercise [224,225]. By participating in the interaction between skeletal muscle and adipose tissue, myokines have the potential to balance and counteract the activity of pro-inflammatory adipokines (see Table 1). PPAR-γ coactivator 1-α (PGC-1α) plays an important role in the regulation of skeletal muscle adaptation to exercise, and the levels of this peptide correlate with those of myokines released from exercising muscles [224]. Moreover, these myokines exhibited anti-inflammatory effects and improved glucose tolerance in obese/diabetic animals [224]. Exercise can also influence the release of adipokines from the adipose tissue of obese individuals by decreasing TNF-α, visfatin, omentin-1, and leptin levels, and increasing APN levels [226].

Like adipose tissue, skeletal muscle has also been shown to release various miRNAs, an additional component of the communication between adipose tissue and muscle [227,228]. New evidence suggests that exercise is also mediated by extracellular vesicles, which contain both classical myokines and other bioactive molecules, including miRNAs [228,229]. APN, which is reduced in visceral obesity and whose release is stimulated by exercise, regulates the number of miRNAs in adipose tissue [230,231]. MiR883b-5p, which is upregulated by APN and lowered in obesity, showed an inhibitory effect on lipopolysaccharide (LPS)-binding protein and Toll-like receptor 4 (TLR4) signaling, thus acting as an important mediator of the anti-inflammatory activity of this adipokine [230].

It has recently been suggested that the penetration of adipose tissue into the muscle also plays a key role in tumor promotion [232]. Epidemiological studies have shown that regular physical activity is associated with reduced development and progression of cancer [233,234]. Animal studies have shown that exercise is associated with reduced tumor growth and metastatic spread [235]. In addition to reducing inflammation, myokines also play a direct role in the tumor-suppressing effects of exercise [194]. Two anti-tumor myokines, OSM [207] and SPARC [236], have recently been identified that inhibit colon tumor formation and inhibit breast cancer cell growth, respectively. OSM has been shown to exert significant in vitro apoptotic effects on tumor cell lines by inhibiting proliferation in a variety of tissues including breast epithelial, melanoma, ovarian, and lung cells [207]. SPARC is secreted into the bloodstream in response to exercise, and its release was found to be associated with the inhibition of colon tumor formation via the increasing of apoptosis [236]. A single training session quickly raised SPARC levels in the blood plasma and muscles, suggesting that contracting myocytes release this myokine into the systemic circulation. This exercise-induced increase in SPARC appears to be muscle specific as no increase in this myokine has been observed in other organs [236]. There are strong indications for a role of irisin as an anti-cancer agent because this myokine has inhibited the viability of several types of cancer cells, including esophageal cancer cells [237,238,239,240]. Exosomal miRNAs have been shown to play an important role in regulating tumor progression and the anti-tumor effects of exercise can be mediated by altered miRNA expression, as suggested recently [241,242].

## 5. Molecular Alterations in Experimental and Clinical BE and EAC Complicated by Obesity

Increased risk of cancer associated with obesity may be attributed to various interdependent mechanisms, such as systemic inflammation, immune dysregulation, adipokine secretion, insulin and insulin-like growth factor1 (IGF-I) signaling, tumor angiogenesis, and the gut microbiota. In addition, optional interventions, such as restriction of diet and exercise, can be prophylactic or therapeutic for obesity and gastrointestinal cancers, including BE and EAC [247]. Recent evidence indicates that adipokine expression and the ratio of leptin to adiponectin are important for metabolic characteristics in patients with esophageal disorders. In addition to an unregulated leptin/adiponectin ratio, the risk of esophageal cancer among obese individuals can be partly explained by several factors: high incidence of GERD, the linear relationship between central obesity and the development of BE, as well as low levels of adiponectin and high levels of leptin. These factors may influence the processes of cell proliferation, the state of insulin resistance that creates the oncogenesis environment, and changes in intestinal and esophageal microbiota due to unhealthy eating habits that promote carcinogenesis [32]. As mentioned, low levels of adiponectin and high levels of leptin, as well as leptin OB receptors, are highly expressed on esophageal epithelial cells. The observation that patients with BE had higher levels of leptin in the fundus than those with normal esophagus confirms that hormones causing metabolic changes may play an important role in the pathogenesis of this disorder due to leptin-mediated signal transduction in BE [243]. Moreover, ObR expression was increased in esophageal epithelial cells. In line with this finding, serum adiponectin was found to be inversely related to BE, particularly in men. The same trend was observed in patients with GERD and erosive esophagitis as decreased levels of esophageal adiponectin and low serum adiponectin levels were reported compared with patients without GERD. Similarly, such an imbalance between leptin and adiponectin was reported to increase the risk of erosive esophagitis [243].

Another axis that could be modified through a lifestyle intervention might be insulin/IGF-1 signaling directly on the esophageal tissue affected by Barrett’s lesions. The molecular changes in the insulin/IGF-1 axis still need elucidation, but insulin resistance is known to create a neoplastic environment. Arcidiacono et al. [248] provided data on esophageal protein expression suggesting that BE patients who entered the intervention program and made lifestyle changes presented with a downregulation of most proteins involved in insulin-/IGF-1-induced molecular signal transduction. These patients not only lost body weight, normalized their glycemic status, improved their HOMA-IR indexes, and decreased their IGF-1 serum levels, but also exhibited lower IGF-1/Binding protein 3 molar ratios [248]. In addition, the molecular analysis of BE tissue revealed a significant reduction in expression of insulin receptor signal1 (IRS1), p70S6K, and the extracellular signal-regulated kinase (ERK1/2) total protein, accompanied by a decrease in IGF-1 serum levels. Furthermore, patients who showed a lower expression of IRS1 belonged to two distinct subpopulations, with one of them displaying a significant decrease in the expression of major proteins involved in insulin/IGF-1 signal transduction such as Akt, p70S6K, and ERK1/2. However, among the second subpopulation, a significant increase in the relative inhibitory phosphorylation of the anti-tumor protein IRS1 and TSC2 and increased activation of the mitogenic pathway associated with ERK1/2 were observed [248]. The interventional lifestyle modification program in these patients resulted in no weight loss, an increase in blood glycaemia and serum leptin, and a decrease in the serum IGF-binding protein 3.

The interventional lifestyle modification program in these patients resulted in no weight loss, an increase in blood glycaemia and serum leptin, and a decrease in the serum IGF-binding protein 3. Interestingly, moderate exercise was beneficial because glucose homeostasis, glycemic control, insulin resistance, and insulin sensitivity improved and reduced IGF-1 availability was observed, especially in those patients who responded optimally to this approach, confirming the possibility of decreased risk of BE evolution towards EAC. Changing eating habits, combined with moderate exercise, resulted in molecular modifications of the insulin/IGF-1 pathway in the esophageal tissue affected by precancerous lesions, ultimately having a beneficial effect in BE patients [248].

Clinical observations that leptin can exert pathological effects by promoting EAC were confirmed by an in vitro study of the EAC cell line OE33 derived from BE [173]. These authors reported that leptin stimulates the proliferation of OE33 cells in a dose-dependent manner while inhibiting cell apoptosis [173]. Expression of long and short leptin receptors by OE33 cells in their study [173] was confirmed by qRT-PCR, Western blotting, and immunocytochemistry. The expression of cyclooxygenase (COX)-2-derived prostaglandins (PG) was considered a potential target enzyme responsible for these effects, as the leptin effect was replicated by the addition of prostaglandin E2 (PGE_2_) and leptin-stimulated cell proliferation resulted in the production of PGE_2_ [173]. Consequently, the deleterious effect of this combination of leptin and PGE_2_ was abolished by the antagonist EP-4 AH23848. Interestingly, the activation of ERK, p38 MAPK, phosphatidylinositol 3‘-kinase/Akt, and Janus tyrosine kinase (JAK)-2 was a result of COX-2 induction, while epidermal growth factor receptor (EGFr) and c-Jun NH2-terminal kinase (JNK) were down-stream targets of COX-2. Moreover, they found that PGE_2_ stimulates JNK phosphorylation in an EGFr-dependent manner, and that activation of EGFr requires protein kinase C, src, and matrix metalloproteinase activity. The subsequent PGE_2_-mediated transactivation of EGFr and JNK appears to be crucial for leptin-induced cell proliferation and this mechanism may contribute to the increased risk of EAC in obesity [173].

Signal transduction and the molecular pathways of visceral obesity affecting the esophageal mucosa remain unexplored. In another study, the authors aimed to identify the pathways by which visceral fat influences oncogenesis [249]. In their study, the expression of ObR and adiponectin 1 and 2 receptors (Adipo-R1, Adipo-R2) was quantified by qPCR and in the human esophageal adenocarcinoma cell line OE33 in vitro [249]. Most of the ObRs expressed in tumors also have expressed Adipo-R1 and Adipo-R2. Despite upregulation of ObR and Adipo-R2 mRNAs, the expression of AdipoR1 mRNA was decreased in more than 50% of the samples. These molecular discoveries were significantly related to the anthropometric and radiological measurements of obesity. Thus, Howard et al. [249] concluded that obesity is associated with an increased expression of ObR and Adipo-R2 in esophageal adenocarcinoma, suggesting that adipocytokine pathways play a pivotal role in the formation of esophageal neoplasms.

Although the role of leptin in promoting the BE cascade to EAC is well documented, the potential influence of another gastric orexigenic peptide, ghrelin, on the progression of BE carcinogenesis has not been extensively studied. In order to investigate the role of ghrelin in the progression of BE, Konturek et al. [246] investigated the expression of adiponectin and ghrelin receptors in the BE OE-19 cell line and in normal squamous epithelium by qRT-PCR method, as well as the effect of adiponectin and ghrelin on apoptosis in BE cells (Bax and Bcl-2 expression) and the effect of ghrelin on IL-1β and COX-2 expression in these cells incubated with TNF-α in vitro. They found [246] that adiponectin enhanced apoptosis, and this effect was accompanied by increased Bax expression and decreased expression of Bcl-2. In contrast, ghrelin failed to affect the apoptosis of OE-19 cells incubated in neutral or acidified medium with or without incubation with deoxycholic acid. The mRNA expression of adiponectin receptors (both, Adipo-R1, and Adipo-R2) was downregulated, while expression of the ghrelin receptor (GHS-R1a) was upregulated in BE cells [246]. Moreover, they observed [246] a decrease in COX-2 and IL-1β expression induced by TNF-α in OE-19 cells when these cells were incubated with ghrelin. The authors [246] concluded that both adiponectin and ghrelin inhibit BE carcinogenesis through two different mechanisms, namely, the adiponectin-induced increase in apoptosis and the anti-inflammatory effect induced by ghrelin. Thus, obesity causing the levels of these two peptides to drop may partially explain the progression of BE into EAC in obese subjects.

It should be noted that visceral obesity is known to increase the local visceral fat tissue, known as the esophagogastric junction fat pad, which may be a source of pro-inflammatory adipokines reaching the mucosa of the distal part of the esophagus at a higher concentration than other tissues [66].

This observation in the cell line in vitro was partially confirmed by the clinical determination of the expression of adipokine receptors in BE and normal squamous epithelium in the same patients along with the correlation of their findings with the measures and parameters of human obesity [168]. In their study, the expression of the adiponectin 1 and 2 receptor protein (Adipo-R1 and Adipo-R2) and the leptin receptor protein (ObR) in biopsies with 27 BE patients and normal squamous epithelium in the same patients as well as in obese subjects and normal controls were evaluated by Western-blot analysis and then confirmed by qRT-PCR to look for particular gene expression. They found that the levels of Adipo-R1 and ObR, confirmed by quantitative mRNA expression, were similar in BE mucosa and squamous epithelium in the same patients. Using linear correlation analysis, a positive correlation was found between Adipo-R1 expression in BE epithelium compared to squamous epithelium in the same patients and between ObR expression in BE and normal epithelium. Adipo-R1 and ObR protein levels were significantly higher in BE patients compared to controls and obese subjects, suggesting that obesity may not be the main cause of deregulation of these peptides, as well as the ghrelin and adiponectin observed in BE, and that overweight may only be to some extent responsible for the induction of adiponectin and leptin receptor expression in BE [168].

The reason for this discrepancy in the results of in vitro and human studies may be related to the influence of two different types of adiponectin that were assessed, namely full-length adiponectin (f-Ad) and globular adiponectin (g-Ad), on the expression of inflammatory factors [250]. The authors investigated the importance of the ROS/NF-κB signaling pathway in adiponectin-regulated inflammation in EAC cells [250]. It is noteworthy that f-Ad and g-Ad differently regulated both mRNA and protein levels of TNF-α, IL-8, and IL-6, yet in a dose dependent manner in OE19 cells. For example, g-Ad increased the production of TNF-α, IL-8, and IL-6 and increased intracellular ROS levels and NF-κB p65 activation, while in contrast, the f-Ad attenuated the production of inflammatory factors and NF-κB p65 activation as well as decreasing the intracellular content of ROS [250].

Apparently, g-Ad exerted a pro-inflammatory effect, while f-Ad caused an anti-inflammatory effect in a ROS/NF-κB-dependent manner in these OE19 cells, suggesting that these two adiponectin forms may exert a different role in pathogenesis of BE progressing to EAC [250].

Travellin et al. [251] examined the morphological, histological, and molecular features of peritumoral and distal adipose tissue in 60 patients with EAC to investigate whether depot-specific differences influence tumor behavior. They confirmed an association between increased adipocyte size, considered as a hallmark of obesity, and leptin expression, angiogenesis (CD31), and lymph angiogenesis (podoplanin); however, these parameters were associated with nodal metastases only in the peritumoral, but not distal, adipose tissue of these patients. In addition, they clearly confirmed an increase in mRNA expression levels of leptin and adiponectin receptors [251]. Furthermore, the mRNA expression of two key regulatory genes of the epithelial–mesenchymal transition (EMT), in particular, alpha-smooth muscle actin (α-SMA) and E-cadherin, was increased in EAC OE33 cells incubated with conditioned medium collected from cultured biopsies of adipose tissue from these patients. This effect was greater in cells treated with the conditioned medium taken, in particular, from the peritumoral adipose tissue of patients with lymph node metastases. It has been concluded that peritumoral adipose tissue secreting depot-specific paracrine factors may directly contribute toward the progression of BE to EAC, and these effects are mediated by leptin [251]. Thus, there is no doubt that dietary factors such as westernized diet can efficiently accelerate the progression of BE to EAC but the mechanisms of these effects are poorly understood.

Recently, the effect of dietary factors, including an obesity-related high-fat diet (HFD), on the progression of BE (called L2-IL1B) to EAC was investigated in an experimental mouse model of esophageal cancer [192]. Interestingly, in that study [192], the L2-IL1B mice were crossbred with mice that express human IL-8 (L2-IL1B/IL8 mice). The esophageal tissues were collected and analyzed for gene expression profiles with qPCR, immunohistochemistry, and flow cytometry. L2-IL1B mice fed with HFD developed esophageal dysplasia and tumors faster than mice fed the control diet. However, it is worth noting that the tumor development rate was independent of body weight [12]. BE tissues collected from L2-IL1B mice fed HFD and L2-IL1B/IL8 mice revealed a substantial number of myeloid cells and cells expressing Cxcr2 and Lgr5 messenger RNAs compared to the control [192]. Mice faeces were analyzed with 16 s ribosomal RNA sequencing and compared to 16 s sequencing data from dysplasia or BE patients. Indeed, the HFD-fed L2-IL1B mice showed accelerated dysplasia and increased levels of cytokines produced in dysplastic epithelium in response to CXCL1 stimulation. Dysplastic changes in mice were accompanied by a change in the intestinal microflora and an increase in the ratio of neutrophils to NK cells in the esophageal tissues compared to the control group [192]. Similar differences were observed in BE patients who experienced EAC compared to patients who did not progress BE to EAC. Thus, evidence has been provided that dietary factors such as HFD promote dysplasia by altering the esophageal microenvironment and the gut microbiome, thereby triggering inflammation and stem cell expansion independent of obesity. HFD promotes dysplasia through the esophageal microenvironment and changes the gut microbiome, leading to inflammation and stem cell expansion independent of obesity [192].

In another study [252], the gene expression analysis of Barrett’s metaplasia and matched normal mucosa from squamous esophagus and gastric cardia was evaluated in BE patients using HG-U133A Affymetrix chips on fresh frozen tissue. Their transcriptome analysis revealed more than 1300 genes expressed in BE, with the exception of single genes such as SOX and PROM1, which were only dysregulated in BE compared to reference tissues [252]. This study [252] provides further evidence of the complexity of understanding the functional molecular changes in gene expression involved in BE development and unveils insights into new molecular pathways that may lead to better therapeutic options and potential targets for future more effective therapy of BE progressing toward EAC.

## 6. Conclusions

Both obesity and EAC rates have increased sharply in recent years in the United States and Western Europe. EAC is a classic example of obesity-related cancer, with the risk of EAC increasing as BMI increases. Pathologically altered VAT in obesity appears to play a key role in this process. Visceral obesity may promote EAC through direct effects on GERD and BE, and reflux-independent effects, including adipokines and insulin resistance. Deregulation of adipokine production, such as an altered leptin to APN ratio, is involved in the pathogenesis of BE and EAC. The limited molecular findings presented to date have underlined a transcriptional feedback loop linking epigenome dysregulation and metabolic alterations in BE and EAC, suggesting that the blocking of this feedback loop seems to be a favorable potential therapeutic strategy in experimental models of BE in vivo and in vitro as well as in high-risk human subjects suffering from these esophageal pathologies. We recommend that lifestyle interventions to increase regular physical activity may be helpful as part of primary BE and EAC prevention. Although many studies have documented the relationship between obesity and the risk of EAC, and the role of risk-modulating non-pharmacological lifestyle interventions, such as the introduction of physical activity as a preventive measure, the mechanism(s) of exercise’s effect on esophagus physiology and pathology still require further explanation in clinical and translational research.

## Figures and Tables

**Table 1:** The role of mediators secreted by adipose tissue (adipokines) and muscle tissue (myokines) in the development of Barrett’s esophagus (BE) and esophageal adenocarcinoma (EAC). BE—Barrett’s Esophagus; EAC—Esophageal Adenocarcinoma; GERD—Gastroesophageal Reflux Disease; IL-1β—Interleukin 1 Beta; IL-6—Interleukin 6; TNF-α—Tumor Necrosis Factor-α. Labelling “↓” means “decreased” while labelling “↑” means “increased”.

Mediator		Role in BE	Role in EAC
Adipokines	Leptin	↑ Pro-inflammatory cells activation [167]; ↑ Pro-inflammatory cytokines production [167]; ↑ Proliferative and invasive capacity of BE cell lines [157,158,159,160,161,162,163,164]; High expression of the leptin receptor in BE cells [103]; Serum levels positively associated with BE [243]; High serum levels considered to be an independent risk factor for BE development [145,158,159,163,164,176,177].	↓ Apoptosis in EAC cells [152,173]; ↑ Proliferation in EAC cells [152,173]; High leptin receptor expression in EAC cells [103]; High serum levels and insulin resistance in BE patients considered to be an independent from GERD risk factor of EAC [163].
	TNF-α, IL-1β, IL-6	Pro-inflammatory effects [66]; Impairs the integrity of the esophageal barrier [66].	↑ Oncogene expression [32]; ↑ Tumor growth and metastasis [244]; ↑ Oxidative damage [245].
	Adiponectin	Anti-inflammatory effects [178]; Lower serum levels in BE patients than in healthy controls [160,161,162]; High receptor expression associated with less advanced disease stage and improved overall survival [103].	Anti-tumor effects [178]: ↑ Apoptosis of EAC cells [246]; ↓ Cancer-promoting effects of leptin in experimental models [157,158,159,160,161,162,163,164,182]. Inhibits grow factors [32]; Low serum levels considered to be an independent risk factor for EAC [160,161,162]; High receptor expression associated with less advanced disease stage and improved overall survival [103].
Myokines		Influence the release of adipokines [226]: ↓ Leptin, TNF-α, visfatin, omentin-1; ↑ Adiponectin. Anti-inflammatory effects [224].	Tumor-suppressing effects [207,236,237,238,239]; ↑ Apoptosis of cancer cells [207,236,237,238,239]; ↓ Viability and proliferation of cancer cells [207,236,237,238,239].

## Data Availability

Not applicable.
